# Disparity between Perceptual Fall Risk and Physiological Fall Risk in Older Cannabis Users: A Pilot Study

**DOI:** 10.3390/ijerph19010109

**Published:** 2021-12-23

**Authors:** Craig D. Workman, Jacob J. Sosnoff, Thorsten Rudroff

**Affiliations:** 1 Department of Health and Human Physiology, University of Iowa, Iowa City, IA 52242, USA; craig-workman@uiowa.edu; 2 Department of Physical Therapy and Rehabilitation Sciences, University of Kansas Medical Center, Kansas City, KS 66160, USA; jsosnoff@kumc.edu; 3 Department of Neurology, University of Iowa Health Clinics, Iowa City, IA 52242, USA

**Keywords:** balance, cannabis, older adults, fall risk, cognition

## Abstract

Aging is associated with cognitive decline and increased fall risk. Cognitive impairment is associated with cannabis use, which is increasing among older adults. Perceptual and physiological fall risk are discordant in some older adults, but whether cannabis use influences this association is unknown. The purpose of this study was to investigate possible disparities between perceptual and physiological fall risk in older cannabis users. Eight older medical cannabis users and eight sex- and age-matched non-users provided data on perceptual and physiological fall risk. Group differences were assessed, and perceptual fall risk was correlated with physiological fall risk. Perceptual risk and most of the physiological fall risk variables were equivalent between the groups. However, cannabis users performed significantly worse on unipedal stance than non-users. In addition, perceptual fall risk had weak correlations with physiological fall risk in the users (Spearman’s *rho* = 0.17–0.41) and moderate-strong correlations in non-users (*rho* = −0.18–0.67). Cannabis users might have a discrepancy between perceptual and physiological fall risk. Because both concepts play a role in quality of life, identifying strategies to improve them may have significant benefits. Future studies investigating additional perceptual (e.g., cognition, fear of falling, depression, anxiety), physiological (e.g., more challenging static and dynamic balance conditions), and general fall risk are warranted.

## 1. Introduction

Aging is characterized by physiological and social changes that make older adults more vulnerable to chronic disease and geriatric conditions, including cognitive impairment and falls [1]. Unintentional falls are common for older adults and a major cause of mortality and morbidity linked with disability and a decline in functional status [2]. Although falls are traditionally associated with deteriorations in physical function, a connection between falls and cognition is emerging. For instance, the annual fall incidence of older adults with moderate to severe cognitive impairment is ~60–80% [3], which is twice that of cognitively normal older adults [4]. Importantly, cannabis use is also associated with impaired cognition [5] that may endure into older adulthood [6,7,8,9]. Additionally, regular cannabis use has been reported to negatively influence cognitive-motor performance and the cerebral mechanisms associated with coordinated movement [10,11].

The most abundant components in *Cannabis sativa* are Δ-9-tetrahydrocannabinol (THC) and cannabidiol (CBD) [12] THC exhibits psychoactive properties and negatively impacts cognitive and executive function [13,14]. CBD is not intoxicating and might be anxiolytic [15], anti-inflammatory, and neuroprotective [16]. The prevalence of cannabis use among adults ≥50 years has increased significantly [17] and rigorous evaluations of the benefits and risks of cannabis use in this population are necessary. While the benefits of cannabis for medicinal use are emerging, e.g., for chronic pain [18], the risks for older adults have not been well defined. Furthermore, cannabis has a known association with cognitive dysfunction [14] and chronic use might exacerbate existent cognitive impairment in this population and further increase fall likelihood. However, few studies have investigated how cannabis use influences fall risk in older adults and investigations on this topic are still nascent.

Perceived fall risk and objective/physiological fall risk (e.g., performance on a balance task) are incongruent in some older adults. Specifically, Delbaere et al. [19] highlighted such a disparity in a cohort of community-dwelling older adults. Aligning fall risk perception with physiological fall risk is important for fall prevention and rehabilitation. For example, challenging balance exercises in people with high perceived risk and low physiological risk might reinforce concerns about falling and reduce engagement in the training. On the contrary, approaches aimed at increasing self-efficacy and fall management might be beneficial for people with greater physiological risk but are less likely to be effective in those who do not self-perceive this high fall risk. Importantly, if and how cannabis use influences the association between perceived fall risk and physiological fall risk is unknown. This is a critical topic to study because cannabis, especially THC-dominant strains, may alter cognition (as mentioned above) and lead to a disparity between perceptual and physiological fall risk.

In our recent study [20], we reported that older cannabis users had a higher likelihood of falling than older non-users. However, this analysis did not address whether differences in perceptual or physiological risk, or both together, might help explain this finding. Therefore, the purpose of this pilot study was to report the findings of a follow-up analysis of this dataset [20] to investigate possible discrepancies between perceptual and physiological fall risk in older cannabis users. It was hypothesized that older cannabis users would exhibit a poor perception of fall risk and have a higher physiological fall risk than non-users, indicating a disparity between the two concepts in the users group.

## 2. Materials and Methods

The methodology has been detailed previously [20] and only the information relevant for the present analysis will be addressed below. Importantly, the conditions/symptoms purportedly amenable to medical cannabis use are eclectic, with pain control as the most common [21,22]. Given the novelty of investigating fall risk in older cannabis-using adults, our previous study [20], and by extension the present follow-up analysis, was broadly inclusive of all older cannabis users and did not emphasize or focus on a particular subgroup of users (e.g., those with chronic pain). Therefore, eight community-dwelling medical cannabis users (Users; median [range] = 60 (52–66) years) and eight sex- and age-matched controls (Non-Users; 61 (53–66) years; see Table 1 for additional subject characteristics) were recruited according to the following inclusion criteria: (a) 50–80 years of age, (b) healthy enough to complete the protocol based on information obtained from a clinical exam and past medical history, (c) part of the Iowa Medical Cannabidiol program (Users; any subjects with an approved condition for medical cannabis use were included) or have not used cannabis for ≥5 years (Non-Users), (d) able to comprehend the protocol, as indicated by the ability to respond to questions about the study after reading the consent form, (e) able to use and be contacted by telephone, and (f) able to speak, read, and understand English in order to complete a questionnaire in English. Exclusion criteria were: (a) pregnancy, (b) history of traumatic brain injury, and (c) other drug use or alcoholism. This study was approved by the University of Iowa’s Institutional Review Board (IRB#201909808, approved on 17 December 2019), and all subjects provided written consent before participating as per the Declaration of Helsinki.

### 2.1. Experimental Protocol

Subjects completed one session. After signing the consent form, a urine test (iScreen IS1THC dipstick; Alere Toxicology, Portsmouth, VA, USA) was conducted to detect the presence of cannabis and verify group assignment (Users vs. Non-Users). Subjects then completed item 1 of the Activities Balance Confidence scale (ABC-1; perceived fall risk) and item 14 of the Berg Balance Scale (BBS-14; physiological fall risk) as part of the fall risk model developed by Lajoie and Gallagher [23]. ABC-1 asks subjects to rate their balance confidence, from 0% to 100%, when walking around their house [24] and BBS-14 asks subjects to stand on one leg for 10 s and is rated on a 0 (“unable to try or needs assist to prevent fall”) to 4 (“able to lift leg independently and hold for >10 s”) scale [25]. ABC has an excellent correlation with the Fear of Falling Avoidance Behavior Questionnaire (*r* = 0.678, *p* < 0.01) [26] and the BBS (*r* = 0.752, *p* < 0.01) [27]. Static posturography (physiological fall risk) was also performed on a balance board (Balance Tracking Systems, San Diego, CA, USA). For this task, the subjects stood on the balance board for 60 s with their arms folded and their eyes looking at a symbol placed ~0.9 m in front of them at eye level. The primary outcomes included the center of pressure (COP) path length in the anterior-posterior (AP-Path) and medial-lateral (ML-Path) directions, and the area of an ellipse that encapsulates 95% of the 2D area explored (COParea). These data were automatically calculated and displayed within the balance board software and recorded on a data collection sheet. The same investigator performed and scored all the measurements for each subject.

### 2.2. Statistical Analysis

Outcome variables were tested and visually inspected a priori for normality with the Shapiro-Wilk test and Q-Q plots. The normality assessment revealed that BBS-14 and COParea did not meet the normality assumption. Therefore, group differences for these variables were tested with the Mann–Whitney U test and the common language effect size indicator (A). The effect size *A* is an appropriate effect size for non-parametric analyses [28,29] and signifies the probability that a random datum from one group will be larger/smaller than a random datum from the other group. The value of *A* ranges between 0.0 and 1.0, with 0.5 construed as no effect (i.e., 50% probability) and either extreme (0.0 or 1.0) as complete separation of the groups. The remaining normally distributed variables were analyzed with independent t-tests, accompanied by Cohen’s d effect size (d < 0.2 = small, 0.5 = medium, >0.8 = large). Correlations (Spearman’s *rho*; more conservative statistic, considering the small sample size) between ABC-1 and the physiological fall risk outcomes (BBS-14, AP-Pathlength, ML-Pathlength, and COParea) were also calculated. The interpretation of correlations is highly variable and somewhat arbitrary [30]; nevertheless, common cut-offs of *rho* < 0.20 weak, 0.4 = moderate, >0.6 = strong were judged as appropriate for the context of the current analysis [30]. Significance was accepted at *p* < 0.05 and analyses were performed with GraphPad Prism 9.0 (GraphPad Software, San Diego, CA, USA). Given the exploratory nature of this pilot analysis, the *p*-value was not corrected for multiple tests.

## 3. Results

**Figure 1:**
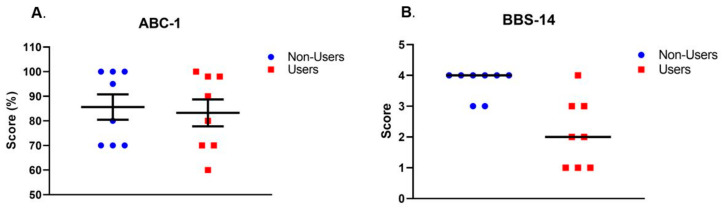
(**A**) Activities-Specific Balance Confidence (ABC) scale score for Item 1 (walking around the house). A higher score indicates higher balance confidence. The difference between the cannabis Users and the Non-Users was not significant (*p* = 0.76, Cohen’s d = 0.1). The data are mean ± SEM. (**B**) Berg Balance Scale (BBS) score for Item 14 (stand on one leg). A higher score indicates better balance performance. The difference between the cannabis Users and the Non-Users was significant (Mann–Whitney *U* test: *p* = 0.008, Common language effect size *A* = 0.89). The bar represents the median score, and *A* represents the probability that a random datum from the Non-Users will be larger than a random datum from the Users (i.e., 89% probability).

All subjects completed the testing as detailed above. As previously described [20], the results of the analysis indicated that perception of fall risk (i.e., ABC-1; Figure 1A) and most of the measures of physiological fall risk (i.e., static posturography measures; Table 2) were not significantly different between the Users and the Non-Users (*p* = 0.28–0.76; see Table 2 for effect size). However, the score on BBS-14 revealed that the Non-Users had a better balance performance than the Users (*p* = 0.008, *A* = 0.89; Figure 1B). Furthermore, the magnitude of the correlations of the physiological balance measures with ABC-1 were generally weak for the Users, while 2/4 of the correlation magnitudes were moderate–strong for the Non-Users (Table 3); however, none of the correlations in either group attained significance (*p* = 0.20–0.74). Most noteworthy was the difference in the correlations of ABC-1 and BBS-14 between the Users and the Non-Users (Spearman’s *rho* = 0.17 and 0.67, respectively).

## 4. Discussion

To the best of our knowledge, this is the first study that aimed to investigate possible disparities between perceptual and physiological fall risk in older cannabis users. We hypothesized that the effects of chronic cannabis use might further increase that risk and lead to a greater disparity between the perception of fall risk and physiological fall risk. As reported in our previous report [20] the Users had a greater physiological fall risk than the Non-Users. The new finding of this analysis was that despite a significantly increased physiological fall risk (lower scores on BBS-14), the Users had an equivalent perceived risk (ABC-1) and a weaker association between these two factors compared with the Non-Users. Taken together, these results indicate that the cannabis Users might have a disconnect between their perception of fall risk and their physiological fall risk compared to their Non-User peers.

It is well established that cannabis, especially products with a high THC content, negatively impairs cognitive and executive function in healthy adults, which may increase the risk of schizophrenic-like psychosis or other severe mental illnesses [31]. Although CBD purportedly minimizes some of the negative side effects and enhances the therapeutic efficacy of THC [32,33,34], a recent study by Arkell et al. [35] found that a combination of THC and CBD increased plasma concentrations of THC metabolites and subtly increased cognitive impairment more than THC alone. These results may have significant implications for older adults that use cannabis products containing both THC and CBD. Importantly, this population already has an elevated fall risk from cognitive impairment and executive dysfunction associated with normal aging [36].

Performances on cognitive tasks in cannabis users have yielded mixed findings. One study in chronic users showed significant cognitive impairments only at higher doses (≥30 mg THC cigarette) [37], whereas another reported acute impairment at a 17 mg dose of THC, but not a 13 mg dose [38]. A study by Perez-Reyes et al. [39] also found that THC produced acute, dose-response effects on THC plasma concentration, heart rate acceleration, and psychological ratings within the range of 10 mg–20 mg of THC. There was no evidence of cognitive differences between the Non-Users and the Users in the current dataset [20], despite half of the latter group using THC-dominant products. However, there are some important differences between our study and those presented above that might explain these differences: (1) medical cannabis in Iowa has more restrictive dosing (between 5 mg and 20 mg of THC per dose) than most recreational products and may not be potent enough to induce cognitive impairment; (2) the subjects in the studies above used combustible THC products and our subjects used ingestible forms (tablets/capsules), which have a lower THC bioavailability than smoked cannabis [40]; and (3) the small number of subjects using a given THC:CBD ratio product prohibited subgroup analyses, which may have masked any cognitive differences in the Users groups. Nevertheless, this analysis still revealed a disparity between the perception of fall risk and physiological fall risk in the Users compared with the Non-Users. Thus, investigations on if and how chronic use of ingested forms of various cannabis ratios might affect the association of perceptual and physiological fall risk differently are recommended.

Our findings indicated that the fear of falling (ABC-1) in the older Users did not reflect an accurate appreciation for their reduced functional balance capabilities (BBS-14) and their increased likelihood of falling [20]. The implication is that older cannabis users with an inappropriately low fear of falling might take undue balance risks beyond their physical abilities, with potentially injurious consequences. Interestingly, a recent meta-analysis indicated that higher THC doses were associated with higher incidences of thinking and perception disorder symptoms in adults ≥50 years old [41]. In combination with the present findings, these meta-results might indicate that some forms of cannabis adversely influence perceptive balance acuity; however, more research on whether cannabis alters perception is certainly required to substantiate such a claim. Nonetheless, intervention programs should help older users develop a more realistic appraisal of their fall risk or improve physical functioning in concert with fear diminution, rather than focusing solely on ameliorating balance decrements.

There was a lack of difference between the groups in static posturography measures despite different performances on BBS-14. It is possible that the Users had a residual physiological fall risk that was not encompassed by the posturography assessment or that they used ‘stiffening’ compensation strategies (e.g., through co-contraction of antagonist muscles) to minimize the risk of falling during this balance test [42]. Additionally, a quiet stance with the eyes open may not have been a sufficiently challenging condition to elucidate group differences, and more difficult tasks, e.g., eyes closed and/or narrower bases of support, or more sophisticated COP analyses (e.g., entropy-based measures) [43] might have better distinguished the groups. Furthermore, reduced leg muscles muscle strength, which was not measured in this study, could have affected postural stability by limiting their capacity to generate stabilizing torques at the ankle, knee, and hip joints, especially in the challenging BBS-14 condition. It is also worth noting that the BBS has some subjectivity in scoring some of the items. However, BBS-14 has clear, objective cut-offs for the different ratings and is unlikely to be influenced by researcher interpretation.

Two fundamental weaknesses of this pilot study were the small number of participants and the variety of cannabis products utilized by the Users, which may have masked some differences, both of which suggest caution in interpreting the results. Still, one purpose of pilot work is to provide effect size estimations for larger studies; thus, the effect sizes in Table 2 and Table 3 can be used to power future investigations. Similarly, post-hoc power analyses indicated that the power of the static posturography and ABC-1 outcomes were 5–17%, while the power for BBS-14 was 92%. This either suggests that (1) some of these analyses were underpowered, (2) some of the data come from two distributions that are essentially equivalent, or (3) some of these measures are not sufficiently sensitive to detect group differences. Given the matched demographic characteristics and the potential lack of discriminating ability of linear COP measures [43] like pathlength and COParea, a combination of suggestions 2 and 3 seems the most likely. Therefore, future studies might benefit from employing other sensitive, specific, and reliable measures of fear of falling and non-linear/entropy measures [43]. For example, the falls efficacy scale international (FES-I) is an alternative construct that assesses a subjects’ perceived fall risk [44]. Like the ABC, this scale asks subjects to rate their concern about falling across a wide range of activities of daily living and includes different, and potentially more relevant, functional activities (e.g., cleaning the house, shopping, walking on uneven surfaces). Another potential limitation was a lack of an overt cognitive screening (e.g., the Montreal Cognitive Assessment [MoCA]) [45] for study inclusion. However, because our subjects were community-dwelling (many still working) and able to comprehend the study protocol, we are confident that the lack of overt cognitive screening (e.g., via MoCA) did not adversely influence the results. In addition, the medical reasons for cannabis use included seven subjects with chronic pain and one subject with Parkinson’s disease, who might have significantly contributed to the results. Therefore, an exploratory analysis that excluded the Parkinson’s disease subject was performed and revealed that this exclusion did not change the reported findings. Future work might also benefit from investigating the effects and/or associations of anxiety on balance performance by interrogating anxiety levels (e.g., with a visual analog scale) in each balance testing procedure. Additionally, several neuropsychological constructs have been linked with falling or fear of falling—e.g., depression [2,46], neuroticism [47], and attention and executive function [48]. Data measuring these constructs might be used to better clarify the associations between perceptual and physiological fall risk. These measurements are important because the different THC:CBD ratios might have a critical and distinct impact on these affects and moderate perceptual and physiological fall risk. Furthermore, additional objective measures of physiological fall risk (e.g., gait analysis, more challenging posturography conditions, muscle strength testing) would be beneficial for future investigations. More studies on the associations of fear of falling with reduced/limited physical activity, leading to a deterioration in postural control performances, are also suggested. Lastly, there is a lack of understanding surrounding the effects of different ratios of THC:CBD on fall risk and other related factors. In the absence of such knowledge, older adults may unknowingly use cannabis products that increase their physiological fall risk and/or distort their perceptual fall risk. This might make falls in these subjects more prevalent and negatively impact their quality of life and performance of everyday activities.

## 5. Conclusions

The present pilot study found a disparity between perceived and physiological fall risk in older cannabis users. Given that perceptual and objective fall risk each play a role in quality of life, identifying strategies to improve both may be relevant for enhancing overall quality of life. This is an important consideration for future studies, clinicians seeking to mitigate fall risk in those using cannabis for symptom treatment, and older adults in general. These results add to previous work indicating that rehabilitative therapies aimed at improving fall risk should also incorporate evaluations of perceived fall risk. Importantly, future studies should also design and power their studies to distinguish between different THC:CBD ratios when assessing perceptual, physiological, and general fall risk in older cannabis users.

## Figures and Tables

**Table 1:** Subject demographic information. Data are median (range).

Demographics	Non-Users	Users
Sex (M/F)	3/5	3/5
Age (years)	61.0 (53.0–66.0)	60.0 (52.0–66.0)
Height (cm)	167.6 (157.5–185.4)	167.6 (152.4–185.4)
Weight (kg)	80.5 (59–124.7)	94.1 (60.1–127)
Duration of Cannabis Use (years)	n/a	4.5 (0.6–30)
Uses per week (days)	n/a	5.5 (1.0–7.0)
Uses per day (times)	n/a	1.0 (1.0–3.0)
THC Dominant (n)	n/a	4
THC = CBD (n)	n/a	2
CBD Dominant (n)	n/a	1
Multiple Types (n)	n/a	1
Medical reasons for use (n)	n/a	Pain (7), PD (1)

THC = Δ-9-tetrahydrocannabinol, CBD = cannabidiol, PD = Parkinson’s disease. n/a: not applicable.

**Table 2:** Central tendency, variability, significance, and effect size (d or *A*) for the study variables.

Variable Name	Users	Non-Users	*p*-Value	Effect Size
ABC-1 (%)	83.3 ± 15.4	85.6 ± 14.5	0.76	d = 0.2
BBS-14 (score)	2 (1–4)	4 (3–4)	0.008	*A* = 0.89
AP-Pathlength (cm)	2.5 ± 0.8	2.2 ± 0.6	0.47	d = 0.4
ML-Pathlength (cm)	1.1 ± 0.4	0.9 ± 0.3	0.28	d = 0.6
COParea (cm^2^)	1.6 (0.9–3.8)	1.1 (0.4–6.6)	0.38	*A* = 0.36

Data are mean ± SD or median (range). The effect size *A* represents the probability that a random datum from the Non-Users will be larger than a random datum from the Users (e.g., 89% probability for BBS-14). ABC-1 = Activities Balance Confidence scale, question 1; BBS-14 = Berg Balance Scale, Item 14; AP = anterior-posterior; ML = medio-lateral; COParea = area of an ellipse that encapsulates 95% of the 2D center of pressure trace.

**Table 3:** Spearman’s *rho* correlations of physiological fall risk measures with the perception of fall risk (ABC-1).

	Users	*p*-Value	Non-Users	*p*-Value
BBS-14 (score)	0.17	0.70	0.66	0.21
AP-Pathlength (cm)	0.28	0.51	−0.18	0.58
ML-Pathlength (cm)	0.15	0.74	−0.50	0.20
COParea (cm^2^)	0.17	0.70	−0.23	0.70

ABC-1 = Activities Balance Confidence scale, question 1; BBS-14 = Berg Balance Scale, Item 14; AP = anterior-posterior; ML = medio-lateral; COParea = area of an ellipse that encapsulates 95% of the 2D center of pressure trace. Correlations were interpreted as r*ho* < 0.20 weak, 0.4 = moderate, >0.6 = strong.

## Data Availability

Data are available upon request to the corresponding author.
